# Vitrification Solutions for Plant Cryopreservation: Modification and Properties

**DOI:** 10.3390/plants10122623

**Published:** 2021-11-29

**Authors:** Jiri Zamecnik, Milos Faltus, Alois Bilavcik

**Affiliations:** Crop Research Institute, Drnovska 507, 16106 Prague, Czech Republic; faltus@vurv.cz (M.F.); bilavcik@vurv.cz (A.B.)

**Keywords:** cryoprotectant, ultra-low temperature, glassy state, toxicity

## Abstract

Many plants cannot vitrify themselves because they lack glassy state-inducing substances and/or have high water content. Therefore, cryoprotectants are used to induce vitrification. A cryoprotectant must have at least the following primary abilities: high glass-forming property, dehydration strength on a colligative basis to dehydrate plant cells to induce the vitrification state, and must not be toxic for plants. This review introduces the compounds used for vitrification solutions (VSs), their properties indicating a modification of different plant vitrification solutions, their modifications in the compounds, and/or their concentration. An experimental comparison is listed based on the survival or regeneration rate of one particular species after using more than three different VSs or their modifications. A brief overview of various cryopreservation methods using the Plant Vitrification Solution (PVS) is also included. This review can help in alert researchers to newly introduced PVSs for plant vitrification cryoprotocols, their properties, and the choice of their modifications in the compounds and/or their concentration.

## 1. Introduction

The cryopreservation of plant genetic resources aims to ensure the long-term storage of viable and genetically stable plant material at an ultra-low temperature using liquid nitrogen (LN, −196 °C) or liquid nitrogen vapour (LNV, −165 to −190 °C). At these temperatures, plant tissues are preserved in a state where cellular divisions and metabolic activities are minimized [1,2], thus preserving the genetic integrity for a longer duration [3,4]. The process of cryopreservation ensures the viability of plant tissues for a theoretically unlimited period [5].

Vitrification—glass formation without crystallization [6,7]—is one of the basic principles used in plant cryopreservation methods. Only a few plants can form a vitreous state naturally [8]. Most plants cannot vitrify themselves because they lack glassy state inducing substances and/or have high water content. The cryoprotectant used for vitrification should have at least three primary abilities: a high glass-forming ability, dehydration strength on a colligative basis to dehydrate plant cells to induce the vitrification state, and the cryoprotectant concentration used must not result in excessive toxicity to the plants. Despite the toxicity of Plant Vitrification Solution 2 (PVS2), it remains a highly effective vitrification solution for plant shoot tip systems [9].

Vitrification cryoprotective solutions reduce the risk of damage of the organelle structures by avoid forming ice crystals [10]; this is achieved by increasing the cell viscosity to the point at which ice formation is inhibited both inside and outside the cell [11]. Thus, cryoprotective solutions protect cell membranes with a gelatinous fluid and can form a glassy state in the cells, which helps plants survive at ultra-low temperatures. In addition, they can prevent further lethal water loss and maintain the percentage of regeneration after cryopreservation [12].

Cryopreservation through the application of vitrification solutions was first reported in plant cells [13,14,15]. Currently, the vitrification-based methods that use vitrification solutions are considered the most widely applied for plant cryo-biologists [16,17]. There are more than 800 papers on shoot tip cryopreservation using vitrification solutions in the literature [18,19]. In addition, several vitrification solutions with different compositions have been reported, but most of the vitrification-based protocols use only a few key solutions [19,20,21,22].

Cryopreservation procedures are currently available for many essential plant species [23,24,25]. For successful vitrification protocols using Cryo Protective Agent (CPA), all the freezable water must be removed from the cells through the use of Plant Vitrification Solution (PVS) before LN exposure. Furthermore, other steps are essential to the success of cryopreservation protocols such as preconditioning, hardening, pre-loading, loading, osmoprotection with various substances before cryopreservation [26,27] and unloading after cryopreservation [28,29]. PVS treatment time, concentration, temperature, as well as shoot tip size, age, its physiology are also very important [30]. All these points are beyond the scope of this review.

The issues addressed by this review are the comparison of different PVSs, their differences in the concentration of the substances used, or the modification of their composition graphically in tables for a quick orientation. Modification is a way to improve previously used PVSs for new genotypes or genotypes with low regeneration rates. A case study on 13 different plant species using at minimum four different PVSs and their modifications is listed. In addition, a brief overview of cryopreservation methods using the PVSs is also included. We assume that this review will also help better select PVSs and their modifications for plant cryopreservation progress.

## 2. Cryoprotective Substances

The successful cryoprotection by vitrification is based on eliminating the formation of ice crystals and on reducing the toxicity of cryoprotective substances [31]. Cryopreservation is a reversible process, as long as the optimal combination and concentration of cryoprotective solution effective enough to form vitrified plant tissues are used. Vitrification refers to the physical process of supercooling a liquid to low temperatures and finally solidifying into a metastable glass without undergoing crystallization at a applied cooling rate [32]. The basic characteristics of substances used in cryopreservation are summarized in Table 1. The glass transition temperature tends to increase with increasing the relative molecular mass as opposed to the melting point [33]. In this order, the most commonly used cryoprotective substances are glycerol (Gly), dimethyl sulfoxide (DMSO), ethylene glycol (EG), propylene glycol (PG), sucrose (Suc), and sorbitol (Sor), at different concentrations in combination with MS medium [34] to allow sufficient dehydration of plant material and also induce cryoprotective processes to the cells [35]. DMSO was later discovered to be an active cryoprotective substance, the alkaline in the main, a good ligand, easily alkalized by a strong base, and subject to deprotonation. Some key features of cryoprotective substances are mentioned below and in the Table 1.

The cryoprotective substances are classified into several groups according to the way of penetrating the cells: (a) small substances penetrating the cells through the cell wall and plasma membrane, such as EG, PG, Gly and DMSO; (b) substances penetrating only through the cell wall, e.g., oligosaccharides such as sucrose (Suc), sorbitol (Sor), mannitol (Man), or amino acids such as proline (Pro), and relatively low molecular mass polymers such as polyethylene glycol (PEG1000); (c) substances that do not penetrate through the cell walls or the plasma membrane, such as relatively high molecular mass polymers (soluble proteins, polysaccharides and, polyethylene glycol PEG 6000) [36,37,38].

### 2.1. Substances That Can Penetrate through the Cell Wall and into the Protoplast

#### 2.1.1. Glycerol

Gly was used as the first for mouse embryo cryopreservation [39]. Gly was also the first cryoprotectant to see widespread use in human cryobiology [40]. Due to its higher relative molecular mass and viscosity, it penetrates membranes [35,41,42], but slower than DMSO and EG [42]. Gly occurs in plants in relevant quantities. Fifteen crop plants grown under field conditions had leaf concentrations of Gly between 10 and 39 µg g^−1^ wet weight of tissue [43]. Gly is a polar molecule, freely miscible with water and simple alcohols [44]. Gly shows lower toxicity because it has a low ability to penetrate the membrane, which at high concentrations can lead to osmotic shock [45]. Gly concentration used for cryopreservation is dependent on the specific vitrification solution and ranges from 20% (weight in volume, *w*/*v*) [46] to 50% (*w*/*v*) [47].

**Table 1:** Characteristics of commonly used vitrification solutions and their chemical and physical properties in PVS.

	Substances	Abr.	M_r_	T_m_	T_g_	Density	LD_50_
			g mol^−1^	°C	°C	g cm^−3^	
	1	2	3	4	5	6	7
Sulfoxides							
	Dimethyl sulfoxide [48]	DMSO	78.13	18.45	−132.15	1.10	**
Diols							
	Ethylene glycol [49]	EG	62.07	−13	−113.15	1.11	*
	Propylene glycol [50]	PG	76.06	−59	−100.65	1.4	**
	PEG 8000 [51]	PEG	8000	63	54.82	1.21	***
Triols							
	Glycerol [52]	Gly	92.09	19	−83.15	1.26	*
Polyalcohols							
	Sorbitol [53]	Sor	182.17	111.5	−6	1.49	**
Monosaccharides							
	Glucose [54]	Glu	164.16	147	22.85	1.4	*
Disaccharides							
	Sucrose [54]	Suc	342.3	186	59.85	1.587	***
Proteins							
	Bovine serum albumin [55]	BSA	66.5 kDa	69.8	^§^		-
Amide							
	Formamide [56]		45.04	2.55	-	1.13	*
Plant Vitrification Solutions							
	Plant vitrification solution 1 [46]	PVS1	42.48	−41	−122	1.15	-
	Plant vitrification solution 1 [57]	PVS1			−155		
	Plant vitrification solution 2 [46]	PVS2	37.51	−44	−119	1.14	-
	Plant vitrification solution 2 [58]	PVS2			−115		
	Plant vitrification solution 3 [47]	PVS3	56.29	−35.4	−93.9	1.29	-
	Plant vitrification solution 4 [59]	PVS4	73.53	−33.9	−112	1.31	-
	Plant vitrification solution N [58]	PVSN	-	−50	−110	-	-
	Vitrification solution L [46]	VSL	32.97	−41	−125	1.9	-
	Vitrification solution L [46]	VSL+	-	−47	−121	-	-

^§^—the glass transition is depending on the rate of cooling [60];. Abr.—abbreviation; Mr—relative molecular mass; Tg—glass transition temperature; Tm—temperature of melting point equilibrium; LD50—median dose (*dosis letalis* media) * <5000, ** 5001–20,000, *** >20,001 mg kg^−1^, mL kg^−1^ (mouse, rat, rabbit). The data in columns 3–5 are from the citations mentioned in column No. 1 as upper index; data in columns 6–7 are from the safety sheets.

#### 2.1.2. Dimethyl Sulfoxide

Another common cryoprotectant is DMSO [61], which is used, like Gly, not only for freezing both animal and plant tissues but also in the cryopreservation of microorganisms [41]. DMSO enhances the passage of water molecules across the cell wall and cytoplasmic membrane [62]. The uptake dynamics of DMSO, Gly, and sucrose during dehydration of garlic shoot tips displayed a biphasic nature, with an initial rapid influx followed by a slower, gradual increase in DMSO [63], Gly, and sucrose (Suc) [64]. Room temperature increased the membrane permeability in contrast to a temperature close to zero. The reverse efflux pattern during unloading was similarly temperature dependent. DMSO is commonly used in combination with glycol-type compounds as this combination interacts with water and biological materials slightly different way than these components alone. DMSO is hydrophobic at higher temperatures, meaning it is less toxic at lower temperatures, which leads to lower, slower, and easily controllable tissue dehydration and oxidation of sulphide groups [65]. In addition, DMSO is an effective solvent and has a high osmolality. In contrast, it is harmful and may cause somaclonal variability or mutagenesis. Due to the persisting uncertainties regarding mutagenesis [66,67] or high acute and chronic toxicity, as shown in the case of rhesus monkeys [68] and low order phytotoxicity, some current cryobanks do not use vitrification solutions containing DMSO, as a precaution, not denying the fact that DMSO can be an excellent cryoprotective substance.

#### 2.1.3. Ethylene Glycol

EG acts as a dehydration substance before cryopreservation. Due to its low freezing point, EG is used for its rapid penetration into the cells and its ability to block the ice crystal formation. EG in cryoprotective solutions is usually used at a half concentration in comparison with Gly [69,70]. EG is metabolized to oxalic acid, which increases the acidity of the organism and it is harmful. The oxalic acid reacts with the calcium contained mainly in the cell wall and forms insoluble calcium oxalate crystals stored in vacuoles [71]. High concentrations of the calcium oxalate, crystallized in various crystals, form raphides, druses, or others [72]. EG is commonly used in combination with glycol-type compounds as its combination interacts with water and biological materials [73].

#### 2.1.4. Propylene Glycol

PG is also a commonly used liquid in the cryopreservation process. PG is metabolized to oxalic acid in the metabolic process and acts like EG. However, as a cryoprotectant in warm conditions, PG is non-toxic while EG is metabolized to toxic elements [74].

#### 2.1.5. Polyethylene Glycol

PEG is a liquid or solid, depending on the molecular mass (which typically ranges from 300 g mol^−1^ to 10,000,000 g mol^−1^). The chemical properties are almost the same, but forms with different molecular mass (from 600 to 8000 g mol^−1^), and different physical properties are usually used for cryopreservation [75]. PEG used for plant cryopreservation, e.g., PEG 6000 normally has a concentration of 15% (*w*/*v*) [76].

### 2.2. Substances That Can Penetrate through the Cell Wall

High levels of carbohydrates and sugar alcohols occur in plants as natural, non-toxic cryoprotective substances [77]. Monosaccharides are readily dissolved in cryoprotective solutions and can vitrify plant tissues at a lower concentration level than disaccharides. Therefore, the disaccharide sucrose is often used as an antifreeze agent. Compared to cryoprotection using monosaccharide glucose, sucrose has a higher efficiency [78]. In addition, carbohydrates contribute to the dehydration of samples and are added to the cryoprotective mixture to increase the protection of the membrane integrity in a dehydrated state [70,79,80].

#### 2.2.1. Glucose

Glucose belongs to a group of monosaccharides that reducing sugar due to the presence of an aldehyde group, which is oxidized to a carboxylic acid group to form D-gluconic acid [81]. On the contrary, reducing the aldehyde group of glucose to the primary alcoholic group forms D-Glucitol, called sorbitol. D-Glucose monohydrate is produced in green plants during the photosynthesis process, which is a fast and basic energy supply. Due to its molecular size, it penetrates the cell membrane faster than sucrose, but in an experimental comparison of plant regeneration after cryopreservation, sucrose, as well as the sugar alcohol mannitol, proved to be more useful [69].

#### 2.2.2. Sorbitol

Sorbitol is used in cryopreservation because of its lower melting point [82]. It provides less energy than sucrose (1 g of sorbitol gives up to 10,886 kJ of energy). Göldner et al. [83] introduced a range of carbohydrates to increase the frost resistance of plants (*Digitalis lanata*) used for plant pre-cultivation. They showed that the most damaged plant cells were cultured on sorbitol and proline medium. In contrast, the smallest cell damage occurred when sucrose was used. The concentration of 0.4 M and 0.8 M sorbitol in the pre-cultivation embryogenic tissues of hybrid firs (*Abies alba* × *A. cephalonica*, *Abies alba* × *A. numidica*) has been found acceptable for subsequent survival and regeneration of the plants [84]. High levels of sugars and sugar alcohols are found in many polar plants, insects, fungi, etc., as non-toxic cryoprotectants [85].

#### 2.2.3. Sucrose

Sucrose, composed of fructose and glucose molecules, is the most widespread disaccharide. It is easily hydrolyzable, dissociated by glycosidase invertase to the laevorotatory glucose and dextrorotatory fructose. These translocated sugars are photosynthetically metabolized in the Calvin cycle. Due to their molecular size, these carbohydrates are preferable for the transport assimilated over long distances. Sucrose is energetically abundant (1 g of sucrose provides 16,747 kJ of energy), and it acts as an energy source in heterotrophic nutrition after plant rewarming during its regeneration. The disaccharide sucrose is more effective than the monosaccharide glucose for vitrification [47,86,87,88]. Sucrose is used to promote dehydration before and/or during cryopreservation. Sucrose is normally membrane-impermeable and has low toxicity. The concentration of sucrose used in cryopreservation processes varies from 5% (*w*/*v*) [46] to 50% (*w*/*v*) [47], but most often 40% sucrose (*w*/*v*) [89,90] is used. For mint shoot tips, sucrose reduces the toxicity of ethylene glycol and DMSO at 22 °C and Gly at 0 °C [65].

#### 2.2.4. Amides

Amides are weak cryoprotectants compared to polyols (formamide is too weak to vitrify itself, but can assist vitrification by other cryoprotectants). Adding methyl groups increases the effectiveness of cryoprotectants [91]. Amides, compared to polyols, generally have weak cryoprotective effects [45].

#### 2.2.5. Bovine Serum Albumin (BSA)

BSA decreases the kinetic constant value determined for concentrated EG solutions. However, BSA’s effect was small compared to that which could be produced by a slight increase in EG concentration [92]. On the contrary, Rall [93] suggest that the inclusion of BSA in vitrification solutions may be an effective means of increasing the stability of the amorphous state of vitrification solutions.

## 3. Substances That Do Not Penetrate through the Cell Wall

Substances that do not penetrate through the cell wall are polymers with high molecular weight such as soluble proteins, polysaccharides, mucilage, PEG1000.

Turner et al. [94] proposed that the mode of action of polyalcohols (in our enumeration Gly, mannitol, sorbitol) is not based on molarity, but rather on the total number of hydroxyl (OH) groups present in the medium. Furthermore, based on their results, they propose that the orientation of OH groups is a determining factor in effective cryopreservation [94].

The development of cryogenic technologies is facilitated by biophysical studies capable of monitoring glass stability during cryopreservation [33]. The glass transition temperature of substances depends on the concentration of an aqueous solution, cooling/warming rates, annealing temperature, and type of mixture. For example, three different glass transitions were found in the subzero temperature range of −163, −138, and −93 °C at 20% BSA (*w*/*w*) [60]. Sucrose has also glass transitions at the three different temperatures ranges; T_g1_ (−50 to −45 °C), T_g_′ (−36 °C), and T_g_ (−83 to −57 °C); all sucrose glass transitions are concentration-dependent and the first two are cooling rate-independent [88]. The thermal analysis of plant vitrification solution: PVS1, PVS2, Towill’s, Fahy’s, or Steponkus’ vitrification solutions reveals only a small water peak detected in shoot tips after 120 minutes dehydration duration. Still, recovery of cryopreserved garlic shoot tips exposure to these vitrification solutions was low (from 0 to 25%), in comparison to 80% regeneration after PVS3 [95].

## 4. Vitrification Solutions and Modifications

Many PVS solution variations have been reported to be suitable for cryopreservation of several plants. During their testing, a number of their successful modifications were published. In this section, an attempt is made to give an overview of the most important of them. The original Plant Vitrification Solution 1 (PVS1) was firstly used by Uragami [14] for cultured cells and somatic embryos derived from the mesophyll tissue of asparagus (*Asparagus officinalis* L.). The original composition of PVS1 is in Table 2. 

The PVS1 was used in modifications PVS1-M1 to PVS1-M3 and PVS1-M8 with a lower concentration of DMSO at a concentration up to 6% and in modifications PVS1-M4 with a higher concentration of DMSO (10%). Sucrose was not used in the original PVS1, but it was used at a concentration of 13.7% (*w*/*v*) in the modifications (PVS1-M3 and PVS1-M8). The Gly was in the modification PVS1-M2, PVS-M6, and PVS1-M8 in lower concentrations and higher concentrations in the PVS1-M6 than in the original PVS1. EG was used less concentrated (13% *w*/*v*) in (PVS1-M1 to PVS1-M4) and more concentrated (PVS1-M7) in comparison with the original. PG was used less concentrated in PVS1-M1 to PVS1-M3 and without change in modifications PVS1-M4 to PVS1-M8. Sorbitol was omitted in PVS1-M1 and PVS1-M3 to PVS1-M8. These modifications were also used for shoot tip cryopreservation e.g., *Rauvolfia serpentine* [102] and *Cocos nucifera* L. [103].

Among several PVSs, PVS2 (Table 3) and PVS3 (Table 4) are the most frequently used vitrification solutions. The PVS2 was firstly used at a concentration of 60% [22]. The PVS2 in full-strength [13] (first row in Table 3) is also widely used. Several modifications of PVS2 with different substances and their concentration used have been published: DMSO was used in a lower concentration, from 7.5 to 13% (*w*/*v*), in modifications PVS2-M2 to PVS2-M6 (Table 3). The exact concentration of sucrose (0.4 M) used in the original PVS2 was also in modifications from PVS2-M1 to PVS2-M3 and in PVS2-M10. Sucrose was used in a higher concentration from 15 to 34.2% (*w*/*v*) in the modifications from PVS2-M6 to PVS2-M9 and no sugar was used in PVS2-M4 and PVS2-M5. Gly was used in the modification PVS2-M3, PVS2-M6 in a lower concentration, and PVS2-M8 higher than in the original PVS2. The concentration of EG was unchanged. PG was added in PVS2-M1 and PVS2-M2 at 15 and 7.5% (*w*/*v*), respectively. PEG 8000 was added in PVS2-M3 and PVS2-M10 as 3% (*w*/*v*) solution and PVS2-M6 as 2% (*w*/*v*) solution. Instead of sucrose, sorbitol in PVS2-M5 was added at 15% (*w*/*v*) (Table 3).

PVS2-M1 is a widely used plant vitrification solution [104] in several cryoprotocols for various plant species e.g., *Photinia* × *fraseri* Dress. [105], *Allium sativum* L. [95,106], *Rauvolfia serpentine* [102], *Cocos nucifera* L. [103], *Porphyra yezoensis* [94], *Mentha piperita* L. [65], *Dioscorea* spp. [15]. PVS2-M3 was used for cryopreservation of e.g. *Prunus avium* L. [107], and PVS2-M6 was used for cryopreservation of *Bromus inermis* Leyss [46]. PVS2-M8 was used for cryopreservation of *Clinopodium odorum* [108].

Incubation time in PVS2 varies according to species, temperature conditions, shoot tip size, pretreatment, preculture and, cryopreservation protocol. Therefore, there is no generic time for PVS2. For example, in apple the droplet-vitrification method had the highest regrowth percentage after 30–50 min PVS2 exposure at room temperature [109]; in potato droplet-vitrification had the highest regrowth percentage after 50 min PVS2 exposure at 0 °C [110]; in shallot droplet-vitrification had the highest regrowth percentage after 40–60 min PVS2 exposure at 0 °C [111]; in grapevine droplet-vitrification had the highest regrowth percentage after 90 min PVS2 exposure at 0 °C [112], and in yacon droplet-vitrification had the highest regrowth percentage after 60 min PVS2 exposure at 0 °C [22,113]. DMSO and Gly penetrate the cell wall membrane and increase cellular osmolality avoiding ice formation [7,38,114]. 

Volk and Walters [9] proposed according to their differential scanning calorimeter measure that the PVS2 operates through two cryoprotective mechanisms: (a) it replaces cellular water, and (b) it changes the freezing behaviour of any water remaining in the cells. They expressed the theory that the penetration of some of the components (e.g., DMSO) of PVS2 into the cell is essential to its cryoprotective efficacy. Significantly, the assumption that the mode of action of PVS2 is primarily caused by osmotic dehydration cannot explain its high effectiveness. Cell-penetrating constituents of PVS2 replace water as the cells become dehydrated and prevent injurious cell shrinkage caused by dehydration [9].

**Table 3:** Composition and modification in concentration of substances of Plant Vitrification Solution 2 (PVS2) Sakai [13].

PVS2	DMSO (%)	Suc (%)	Gly (%)	EG (%)	PG (%)	PEG (%)	Sor (%)	Total (%)	Plant
Sakai ^§^	15	13.7	30	15				73.7	*Citrus sinensis* [13]
PVS2-M1		13.7	30	15	15			73.7	*Porphyra yezoensis* [94]
PVS2-M2	7.5	13.7	30	15	7.5			73.7	*Porphyra yezoensis* [94]
PVS2-M3	12.5	13.7	25	15		3 *		69.2	*Prunus salicina* Lindley cv. *Methley x Prunus spinosa* L. [115]
PVS2-M4	15		30	15				60	*Malus* [96]
PVS2-M5	15		30	15			15	75	*Tetraclinis articulata* (Vahl.) [116]
PVS2-M6	13	15	25	15		2		70	*Guazuma crinita* Mart. [117]
PVS2-M7	15	34.2	30	15				94.2	*Poncirus trifoliata* (L.) Raf. × *Citrus sinensis* (L.) Osbeck. [118]
PVS2-M8 ^§§^	15	22.5	37.5	15				52.5	*Allium sativum* L. and D*endranthema grandiflora* [61], *Rubus fruticosus* L. and *Prunus cerasifera* Ehrh [119]
PVS2-M9 ^§§§^	15	15	30	15				75	*Guazuma crinita* Mart. [13]
PVS2-M10	15	13.7	30	15		3 **		76.7	*Populus alba* L. [120]

Important composition changes, added or omitted substances, and/or modification in the concentrations of original PVS2 in % (*w*/*v*) used in the plant cryopreservation. All substances were dissolved in MS medium with 0.4 M of sucrose. The sucrose concentration in PVS2 was approximately 0.15 M. The shaded area expresses no changes concerning the original PVS2. DMSO—dimethyl sulfoxide, Suc—sucrose, Gly—glycerol, EG—ethylene glycol, PG—propylene glycol, PEG—polyethylene glycol, Sor—sorbitol, Total—total concentration of all substances. ^§^ termed ‘100%’ of PVS2; ^§§^ termed PVS2-A3 [61]; ^§§§^—‘60%’ of PVS2; *—PEG 8000 m.w., **—PEG 4000 m.w.

The first use of PVS3 was reported on *Asparagus officinalis* L. by Nishizawa et al. [47]. The original PVS3 plant vitrification solution contained 50% to 50% (*w*/*v*) sucrose and Gly (Table 4).

**Table 4:** The concentration of substances of the original Plant Vitrification Solution 3 (PVS3) [47]. Important composition changes added or omitted substances, and/or modification in the concentrations of original PVS3 in % (*w*/*v*) used in plant cryopreservation.

PVS3	DMSO (%)	Suc (%)	Gly (%)	EG (%)	Total (%)	Plant
Nishizawa		50	50		100	*Asparagus officinalis* L. [47]
PVS3-M1	5	50	50		100	*Porphyra yezoensis* [94]
PVS3-M2		50	30		80	*Malus domestica* Borkh. [69]
PVS3-M3		45	45		90	*Kalopanax septemlobus* [121]
PVS3-M4		40	40		80	*Gentian*, *Wasabi*, *Malus* [22,122] *Fragaria ananassa* [123]
PVS3-M5		60	35	20	105	*Asparagus officinalis* L. [47]

DMSO—dimethyl sulfoxide, Suc—sucrose, Gly—glycerol, EG—ethylene glycol, Total—total concentration of all substances. The shaded area expresses no changes concerning the original PVS3.

Sakai [119] and Benson [11,22] indicated 40% to 40% ratio like an original PVS3. We label this ratio as modification 4 to the original PVS3; this modification is listed as PVS3-M4 in Table 4. Since the first description of PVS3, there have been many reports of vitrification methods using PVS3. PVS3 was used for 136 different plant species till 2007 [22]. The published modifications concern is lowering the concentration of sucrose, mostly in the same ratio, e.g., [22,69,121], to Gly, e.g., PVS3-M3 [121] and PVS3-M4 [22]. PVS3 is the plant vitrification solution without DMSO and EG in the original composition compared to PVS2; curiously, the first modification, PVS3-M1, contains 5% (*w*/*v*) of DMSO in addition to the basic substances. In PVS3-M2, there is only a change in Gly as a 30% (*w*/*v*) concentrated solution [69]. PVS3-M5 contains 20% (*w*/*v*) of ethylene glycol in addition together with an increase of the content of sucrose to 60% (*w*/*v*) and a decrease of glycerol to 35% (*w*/*v*).

The original PVS3 is also widely used in the cryopreservation of *Lithodora rosmarinifolia* (Ten.) [122], *Photinia* x *fraseri* Dress. [105], *Allium sativum* L. [95], *Rauvolfia serpentine* [102], *Cocos nucifera* L. [103], *Bromus inermis* Leyss [103], *Porphyra yezoensis* [94], *Mentha piperita* L. [65], *Dioscorea* spp. [15], *Malus* [96] and other species, but somewhat less than PVS2, the most frequently used vitrification solution [22].

The original PVS4 is without DMSO (Table 5). The two following modifications (PVS4-M1) and PVS4-M2) are with the addition of DMSO. In PVS4-M1 there are only two compounds, DMSO and Gly, at 5% (*w*/*v*); this low concentration of cryoprotectants combined with a slow cooling rate (0.1–0.2 °C min^−1^) act rather as dehydration solution than vitrification solution.

The PVS proposed by Steponkus’ vitrification solution is without sucrose. Sucrose is used in the first modification of Steponkus’ vitrification solution (Steponkus-M1) in the concentration of 13.7% (*w*/*v*). The PVS proposed by Towill’s is slightly modified by increasing the DMSO to 10% and decreasing PEG 8000 to a half. Sucrose is used in the second modification of Towill’s vitrification solution (Towill-M2) in the concentration of 13.7% (*w*/*v*) together with 6.8 % (*w*/*v*) of DMSO influencing the regeneration up to 39% of *Allium sativum* shoot tips [126].

Thermal analysis of PVSs revealed that increasing Gly concentration reduced endothermic peaks, indicating the ice-blocking property of Gly [61]. Increasing sucrose concentration in PVSs also decreased endothermic enthalpies by decreasing explant moisture content and increasing the influx of cryoprotectants [64]. Therefore, balancing the Gly and sucrose concentration in the design of PVSs is also crucial to increase recovery. A limitation of the use of PVS3 is the high osmotic stress increasing during the action; therefore, induction of desiccation tolerance during preconditioning of samples is essential if they are not inherently tolerant [61,128].

## 5. Comparison of Vitrification Solutions on Regeneration

Evaluation of different cryoprotectant solutions and their modification are ordered in Table 6. There is a comparison of three or more different cryoprotective vitrification mixtures.

PVS3, PVS4, PVS5, and Fahy are without modification in Table 6, even though they have several modifications (see Table 3 and Table 4). Among the PVSs, PVS2 and PVS3 are the most frequently used [104]. In some cases, PVS3 may be less toxic to plant species sensitive to PVS2, such as *Allium* sp. [111]. Based on the results presented over the years and also from Table 2, Table 3, Table 4, Table 5 and Table 6, it is evident that the optimum cryoprotectant solution treatment is species or cultivar-specific. Furthermore, the PVSs exposure duration and temperature conditions during incubation are related to the size of the shoot tip, as well as to the preculture and pretreatment conditions [19,135].

The original composition is listed in the first row in each table (Table 2, Table 3, Table 4 and Table 5) and its modifications in concentration and/or in compounds used are followed. Sakai et al. [82] were the first to report a PVS2-vitrification cryopreservation protocol for nucellar cells of *Citrus sinensis*. The modifications of PVS2 were applied on other plants as presented in the paper by Uragami et al. [14] and Maruyama et al. [117].

Modifications of PVS and its influence on the viability of explants have been reported. Suzuki [46] in addition to the three original vitrification solutions (PVS1, PVS2, VSL) (Table 6) presented the effect of 12 other combinations of cryoprotective substances on gentian axillary buds. The best regeneration of 79.7% after liquid nitrogen treatment was achieved with the original VSL. Cho et al. [99] used four original PVSs (PVS1, PVS2, VSL, and VSL+) (Table 6). The best one for *Citrus madurensis* embryonic axes survival after liquid nitrogen treatment was PVS2. Kim [61] modified the PVS2 in nine modifications and PVS3 in four modifications in concentrations of substances in the droplet-vitrification procedure. The best one was the PVS3 without any modifications for shoot tips harvested from in vitro conditions of *Dendranthema grandiflora* T. and garlic clove shoot apices of *Allium sativum* L.

## 6. Vitrification Solution and Cryopreservation Methods

Increased vitrification method efficiency was achieved by treating plants in a pretreatment and preculture steps before cryopreservation of plant shoot tips [22,89,136,137,138,139]. Pretreatment/preculture increases tolerance to PVSs during the dehydration process. Pretreatment conditioning differs by species, and then preculture for some species is crucial [140]. During pretreatments the sucrose intake mostly takes place, and increased content of proline and other protective substances accumulates in the plant shoot tips while growing in the carbohydrate enriched culture medium. The temperature during the incubation of plants in PVSs is important for both toxicity and dehydration. The temperature close to 0 °C for plants treated in PVS2 is crucial and had significantly lower lethality than at 22 °C [65,141,142,143]. When the temperature is subsequently lowered, the penetrating components of PVS2 cryoprotect the cells by restricting the molecular mobility of water molecules and preventing them from nucleating ice crystals [11].

In PVSs vitrification-based methods, most or all of the freezable water is removed by using highly concentrated and viscous cryoprotectant mixtures which, after rapid cooling in LN, form a glass [11,144]. The amount of water in the cells is decreasing due to an accumulation of these substances, and the central vacuole is divided into several smaller ones.

Cryoprotective substances help ensure the stability of membranes and enzymes in subsequent dehydration by vitrification solutions and avoid the formation of ice crystals [145,146]. In this case, the samples are exposed to minutes–a few hours long treatment by several cryoprotective substances before LN exposure. The effect of cryoprotective solution composition for plant regeneration was studied in different plant species [11,61,126,139,147,148]. The published results indicate the importance of PVS compositions, the vitrification protocol, pre-culture, regrowth media, and the application of an appropriate vitrification technique to achieve optimum post-cryopreservation recovery [105,131].

With the combination of the composition of cryopreservation solution (15% DMSO + 3% sucrose) and subsequent slow cooling, a droplet-freezing method was developed for cassava shoot tips [146].

The droplet-vitrification method is derived from the DMSO droplet methods proposed by Kartha et al. [146], and Kaczmarczyk et al. [149] and Schaefer-Menhur et al. [150]. The procedure is similar to the droplet method but with highly concentrated cryoprotective solution PVS2 [104,147,150,151,152] or PVS3 either in original or in its modification PVS3-M4 [122] or both PVS2 and PVS3 [97,113,126] before ultra-fast cooling. Rewarming of the samples is usually done in unloading solution temperated in a 40 °C sterile water bath for 1–2 min. When using potentially phytotoxic DMSO, the cryoprotective mixture is washed out in unloading solution temperated in a water bath with solutions of decreasing concentrations of sucrose or sorbitol as unloading solutions. This method was successfully applied many of plant species and is widely used in genebanks for cryopreserving vegetatively propagated crop collections [153,154].

Other cryopreservation methods use the PVS for inducing vitrification, such as encapsulation-dehydration and encapsulation-vitrification method with PVS2, PVS3 [139,155] foil-vitrification, droplet-vitrification, and droplet-freezing methods with PVS1, PVS2, PVS3, and VSL [105]. In addition other methods use the vitrification solutions PVS2 and PVS3 in V cryo-plate and D cryo-plate methods [27], PVS2 in cryo-mesh method [156,157], and PVS2 in vacuum infiltration vitrification method (VIV) [158].

The determination of plant survival and regeneration level is done by a visual evaluation of growing the plants in vitro conditions. The ratio of regenerated to the total number of cryopreserved plants is expressed as a success of the cryoprotocol (see Table 6).

Cryoprotectants can change the biophysical properties of plant parts. Cryoprotectants are selected based on their potential non-toxicity, high osmolality, and ability to penetrate as a particular component of vitrification solution into the cell. A low survival and regeneration of plants can also be caused by insufficient osmotic adjustment of plant material, excessive shrinkage of cells in hypertonic conditions, the toxicity of the vitrification solution, low penetration ability of the cryoprotective solution into the plant tissue, low dehydration of the plant tissue and subsequent formation of intracellular ice crystals during freezing [6]. During slow cooling, the sample may reduce its cell surface due to the loss of cytoplasmic membrane, and the cell lysis can occur upon returning to a normal state [64,79]. The toxicity of cryoprotective substances can be associated with the denaturation of proteins, which are damaged either by low temperature or high cryoprotectant concentration necessary for plant tissue vitrification. The strongest vitrification is achieved with cryoprotective substances, which can bind hydrogen bonds to water molecules. They make the interaction of hydrogen bonds water-water, which is the basis for forming ice crystal structure. These substances can be bound by hydrogen bonds in proteins, causing their denaturation [35]. Reduction of water bound to proteins can damage the cells. Dehydration to the level of bound water is essential for successful cryopreservation without the use of cryoprotective substances.

The following steps are recommended for cryopreservation of a new plant species. First, the new PVS should be chosen from cryoprotective solutions close to the species family with the highest regeneration ability. The second possibility is to use the PVS widely used for most plants, e.g., PVS2, PVS3, etc. After choosing the PVS, it is necessary to test the toxicity level following the growing test and level of dehydration [159] according to their regeneration. If the thermal analysis is available, it will help a lot at this step [160]. The difference between the regeneration rate of control and ultra-low temperature treated plants is the potential to improve regeneration by improved vitrification solution.

## 7. Conclusions

Cryopreservation methods allow long-term storage of genetically unique plant material in the vitreous state at ultra-low temperatures of LN, which leads to the suppression of all biochemical reactions. Vitrification solutions as a mixture of two to seven substances induce a glassy state in plant tissues and prevent the ice crystal formation during the cooling and warming process. The cryoprotective mixture toxicity can be reduced by an appropriate combination or decrease in the concentration of cryoprotective substances and/or physical condition, mainly low temperature at which those are applied. The best cryoprotective solutions can reduce the toxicity of the vitrification mixture. Easier and faster cryoprotectant penetration into the cells and tissue dehydration to the optimal level for cryopreservation will increase the survival and regeneration of plants and extend cryopreservation methods for other plant species and genotypes. The widely used vitrification solutions meet these demands for high regeneration (over the minimum standard of cryobank) after cryopreservation.

## Figures and Tables

**Table 2:** The concentration of substances of the original Plant Vitrification Solution numbered one (PVS1) uses Uragami [14].

PVS1	DMSO (%)	Suc (%)	Gly (%)	EG (%)	PG (%)	PEG (%)	Sor (%)	Total (%)	Plant
Uragami	7		22	15	15		9.1	68.1	*Asparagus officinalis* L. [14]
PVS1-M1	6		22	13	13			54	*Malus* sp. [96]
PVS1-M2	6		19	13	13		9.1	60.1	*Porphyra yezoensis* [94]
PVS1-M3	6	13.7	22	13	13			67.7	*Allium sativum* L. [97]
PVS1-M4	10		22	13		13		58	*Dioscorea opposite* [98]
PVS1-M5	7		22	15		15		59	*Citrus madurensis* [99]
PVS1-M6		31.1	18.4	15				64.5	*Allium sativum* L. [100]
PVS1-M7	7	15	22	30				74	*Bletila strata* [101]
PVS1-M8	5	13.7	13.7					32.4	*Citrus madurensis* [99]

DMSO—dimethyl sulfoxide, Suc—sucrose, Gly—glycerol, EG—ethylene glycol, PG—propylene glycol, PEG—polyethylene glycol 8000 m.w., Sor—sorbitol, Total—total concentration of all substances. Significant composition changes added or omitted substances and/or modification in the concentrations of original PVS1 in % (*w*/*v*) used in plant cryopreservation. The shaded area expresses no changes concerning the original PVS1.

**Table 5:** Plant Vitrification Solution 4 (PVS4), Steponkus’, Towill’s and their modifications in concentration, composition, and some omitted and added substances in % (*w*/*v*).

	DMSO (%)	Suc (%)	Gly (%)	EG (%)	PEG * (%)	Sor (%)	BSA (%)	CaCl_2_ (mM)	Total (%)	Plant
PVS4		20.5	35	20					75.5	Various plants [124]
PVS4-M1	5		5						10	*Malus* [96]
PVS4-M2 **	10	15	20	30				10	75	*Citrus madurensis* [99] *Bromus inermis* Leyss [46]
PVS4-M3 ***	10	5	20	30				10	65	*Bromus inermis* Leyss [46]
Steponkus				43.5		16	6		65.5	*Secale cereale* L. [125]
Steponkus-M1		13.7		50		15	6		84.7	*Allium sativum* L. [126]
Towill	7.8			35	10				52.8	*Mentha aquatica× M. spicata* [127]
Towill-M1	10			35	5				50	*Guazuma crinita* Mart. [117]
Towill-M2	6.8	13.7		35	10				65.5	*Allium sativum* L. [126]

DMSO—dimethyl sulfoxide, Suc—sucrose, Gly—glycerol, EG—ethylene glycol, PG—propylene glycol, PEG—polyethyleneglycol, Sor—sorbitol, BSA—bovine serum albumin, Total—total concentration of all substances. * PEG 8000 m.w., ** known also as VSL, *** known also as VSL+ [46]. The shaded area expresses no changes concerning the original PVS.

**Table 6:** Regeneration rate (%) after application of Plant Vitrification Solutions and their modifications (M1_M4 for details see Table 2, Table 3, Table 4 and Table 5). Three or more Plant Vitrification Solutions or their modifications at one particular species.

PVS1		PVS2		PVS3	PVS4	PVS5	VSL	Steponkus		Towill		Fahy		Plant
	Mod.		Mod.						Mod.		Mod.		R/S **	
0	M3	0		80	0			0	M1	0	M2	0	R	*Cocos nucifera* L. [103]
11	M1	27		80	25			23		39	M2	11	R	*Allium sativum* L. [126]
0		20		0			0	0 ^§^		0		0	R	*Elaeis guineensis* [129]
0	M1	0	M4	70	0	0							S	*Malus* [96]
36		30		20	28								S	*Centaurea ultreia* [130]
65		75		65	65								S	*Citrus madurensis* [99]
55		32					80						S	*Gentian* [46]
80		20		0			14						R	*Fraser Photinia* [105]
18	M2	24	M1,M2	15									R	*Porphyra yezoensis* [131]
34		49		25									R	*Solanum tuberosum* L. [132]
0	M1	87		0									R	*Rauvolfia serpentina* L. [102]
		59		0	38								R	*Discorea* [15]
92	M1	82		52 ^§§§§^								83 *	S	*Ipomoea batatas* [133]

^§^ according to Watanabe and Steponkus [134]; ^§§§§^ 88% of PVS3; * PVS N (1 M sucrose + 15% glycerol + 14% ethylene glycol [133]; ** R—stands for regeneration, regrowth, S—stands for survival after cryopreservation. The PVS3, PVS4, PVS5 [96], VSL [46], and Fahy’s vitrification solution [31] are unmodified compared to other PVSs.

## Data Availability

The data presented in this study are available on request from the corresponding author.
